# Disitamab vedotin in preclinical models of HER2-positive breast and gastric cancers resistant to trastuzumab emtansine and trastuzumab deruxtecan

**DOI:** 10.1016/j.tranon.2025.102284

**Published:** 2025-01-20

**Authors:** Negar Pourjamal, Vadim Le Joncour, György Vereb, Cilla Honkamaki, Jorma Isola, Jeffrey V Leyton, Pirjo Laakkonen, Heikki Joensuu, Mark Barok

**Affiliations:** a Helsinki University Hospital and University of Helsinki, Helsinki, Finland; b Laboratory of Molecular Oncology, Biomedicum, University of Helsinki, Helsinki, Finland; c Neuroscience Center, Helsinki Institute of Life Sciences (HiLIFE), University of Helsinki, Helsinki, Finland; d Department of Biophysics and Cell Biology, Faculty of Medicine, University of Debrecen, Debrecen, Hungary; e HUN-REN–UD Cell Biology and Signaling Research Group, Faculty of Medicine, University of Debrecen, Debrecen, Hungary; f Laboratory of Cancer Biology, Medical Faculty, University of Tampere, Tampere, Finland; g School of Pharmaceutical Sciences, Faculty of Medicine, University of Ottawa, Ottawa, Canada; h Translational Cancer Medicine Research Program, Faculty of Medicine, University of Helsinki, Helsinki, Finland; i Laboratory Animal Center, Helsinki Institute of Life Science (HiLIFE), University of Helsinki, Helsinki, Finland; j ICAN Digital Precision Medicine Flagship Program, University of Helsinki, Helsinki, Finland; k Department of Oncology, Helsinki University Hospital and University of Helsinki, Helsinki, Finland

**Keywords:** Antibody drug conjugate, Disitamab vedotin, Human epidermal growth factor receptor 2, Trastuzumab deruxtecan, Trastuzumab emtansine

## Abstract

**Background:**

Most HER2-positive breast or gastric cancers eventually become resistant to the approved anti-HER2 antibody-drug conjugates (ADC) trastuzumab emtansine (T-DM1) and trastuzumab deruxtecan (T-DXd). Disitamab vedotin (DV) is a novel anti-HER2 ADC that binds to a different epitope on HER2 compared to trastuzumab. We assessed the efficacy of DV in breast and gastric cancer cell lines and xenografts, including tumor models resistant to T-DM1 and T-DXd. Additionally, we investigated whether combining two anti-HER2 ADCs could enhance the efficacy of the individual ADCs.

**Methods:**

The efficacy of DV, T-DM1, and T-DXd, both as single agents and in combinations, was assessed using an AlamarBlue cell proliferation assay in HER2-positive breast and gastric cancer cell lines, including those resistant to T-DM1 and T-DXd. The efficacy of DV was evaluated also in breast and gastric cancer SCID mouse xenografts that had progressed on T-DM1 and/or T-DXd. ADC combinations were tested in breast and gastric cancer xenografts.

**Results:**

DV was effective in cell lines resistant to T-DM1 and/or T-DXd, and it inhibited the growth of breast and gastric cancer xenografts that had progressed on T-DM1 and/or T-DXd. The combinations of DV plus T-DM1 and DV plus T-DXd showed greater efficacy than the corresponding single agents in both breast and gastric cancer cell lines and xenografts.

**Conclusions:**

DV was effective in treating breast and gastric cancer xenograft tumors resistant to T-DM1 and/or T-DXd. The combination of DV with T-DM1 or T-DXd demonstrated promising activity.

## Highlights

• We compared 2 trastuzumab-based ADCs and 1 hertuzumab-based ADC in xenograft models.
• The trastuzumab based (T-DM1, T-DXd) and hertuzumab-based (DV) ADCs were effective.
• DV inhibited breast and gastric cancer xenografts progressing on T-DM1 or T-DXd.
• Combinations of DV with T-DM1 or T-DXd were more effective than the single ADCs.

## Introduction

The human epidermal growth factor receptor-2 (HER2), overexpressed in 15–20 % of breast and gastric cancers, represents an important therapeutic target [1,2]. Trastuzumab emtansine (T-DM1) and trastuzumab deruxtecan (T-DXd) are trastuzumab-based antibody-drug conjugates (ADCs) designed to selectively deliver a cytotoxic payload to HER2-expressing cells. T-DM1 delivers a maytansine derivative (DM1), while T-DXd delivers an exatecan derivative (deruxtecan) [[3], [4], [5]]. T-DM1 has been approved by the U.S. Food and Drug Administration (FDA) for HER2-positive early and advanced breast cancer [[6], [7], [8]] but not for gastric cancer [9]. T-DXd is approved for HER2-positive and HER2-low advanced breast cancer, as well as HER2-positive advanced gastric cancer [[10], [11], [12]] (Supplementary Table S1). Unfortunately, resistance to T-DM1 and T-DXd eventually develops during the treatment [8,13,14]. Advanced breast and gastric cancers are ultimately lethal, and patients with resistant disease have limited therapeutic options, highlighting the need of new treatment strategies.

In this study, we investigated the efficacy of disitamab vedotin (DV), a novel anti-HER2 ADC, in breast cancer and gastric cancer cell lines and xenografts that are resistant to T-DM1 and/or T-DXd. DV has been conditionally approved by the National Medical Products Administration of China for the treatment of HER2-positive advanced gastric cancer and urothelial cancer [[15], [16], [17]]. DV contains a novel HER2-targeting monoclonal antibody, hertuzumab, which binds to a different epitope on HER2 compared to trastuzumab. DV delivers a monomethyl auristatin E (MMAE) cytotoxic payload into cancer cells [18]. We recently found that T-DXd and DV inhibited the growth of lung metastases more effectively than T-DM1 in a HER2-positive breast cancer lung metastasis model [19].

In the current study, we also assessed the combination of two ADCs to enable the delivery of two distinct cytotoxic payloads to cancer cells. To our knowledge, there has been limited prior research on this strategy. We found that DV was often effective in preclinical models for treating cancer resistant to T-DM1 and/or T-DXd, and that DV shoved promising activity in combination with T-DM1 or T-DXd.

## Materials and methods

### Cell lines

We studied five HER2-positive human breast cancer cell lines (BT-474, EFM-192A, JIMT-1, SKBR-3, and UACC812), one HER2-negative breast cancer cell line (Hs-578T) as a control line, and five HER2-positive human gastric cancer cell lines (OE19, N87, RN87, ROE19, and SNU-216) (Supplementary Table S2). Seven of these cell lines were T-DM1-sensitive (BT-474, EFM-192A, JIMT-1, SKBR-3, N87, OE19, and UACC-812), while three were T-DM1-resistant (RN87, ROE19, and SNU-216) [[20], [21], [22], [23], [24]]. We generated the T-DM1-resistant gastric cancer cell lines RN87 and ROE19 by treating the N87 and OE19 cells, respectively, with increasing concentrations of T-DM1 (Roche Ltd., Basel, Switzerland) [21,22]. The authenticity of all cell lines was confirmed using short tandem repeat analysis. Cells were cultured according to the recommended specifications and tested mycoplasma-free.

### Cell viability assay

The effects of T-DM1 (Roche Ltd., Basel, Switzerland), T-DXd (AstraZeneca, Cambridge, UK), and DV (MedChemExpress, Monmouth Junction, NJ, USA) on cell growth were studied using the AlamarBlue assay (Thermo Fisher Scientific, Waltham, USA) [21,22]. Briefly, the cells were trypsinized and plated in 96-well flat-bottomed tissue culture plates. The efficacy of each ADC as a single agent was tested at concentrations of 0.0001, 0.0006, 0.003, 0.016, 0.08, 0.4, 1, 2, and 10 µg/mL. Next, two ADC combinations were investigated: (1) increasing concentrations of T-DM1 with a fixed concentration of DV, and (2) increasing concentrations of DV with a fixed concentration of T-DM1. The tested concentrations of T-DM1 or DV were 0.0001, 0.0006, 0.003, 0.016, 0.08, 0.4, 1, and 2 µg/mL. The fixed ADC concentration was chosen so that the ADC as a single agent had no or only minor inhibitory effect on cell growth. Further details are provided in the Supplement.

### Mouse xenograft models

To establish xenograft tumors, five- to six-week-old female SCID mice (C.B-17/IcrHan Hsd-Prkdc^scid^, Envigo RMS B.V., Horst, The Netherlands) were injected subcutaneously with 1 × 10^7^ JIMT-1 cells, 9 × 10^6^ N87 cells, or 1.1 × 10^6^ RN87 cells in 100 µL of cell culture medium.

To investigate the effects of the single ADCs, T-DM1 (0.5 mg/kg or 5 mg/kg), T-DXd (5 mg/kg), or DV (0.5 mg/kg or 5 mg/kg) was administered intravenously at 7-day intervals. Three ADC combinations were also evaluated: [1] T-DM1 plus T-DXd (5 mg/kg of each), [2] T-DM1 plus DV (0.5 mg/kg, or 5 mg/kg of each), and [3] T-DXd plus DV (5 mg/kg of each). In the xenograft tumor models, each treatment group consisted of five to six mice unless otherwise specified.

Tumor diameters were measured using a digital caliper, and tumor volume was calculated using the formula T_vol_ = π/6 × larger diameter × (smaller diameter)^2^. A complete response was defined as tumor shrinkage to non-palpable levels, partial response as a tumor volume reduction of ≥ 20 % but < 100 %, and tumor progression as a volume increase of ≥ 20 % compared to the previous measurement, with a minimum size of 1.1 mm³, provided the volume remained stable or increased in subsequent measurements. The general condition of the mice was assessed through visual inspection, weight measurements, and the body condition score [25]. Mice were euthanized if they lost ≥ 20 % of their body weight, the body condition score deteriorated, any tumor dimension exceeded 15 mm, or tumor ulceration occurred.

### Flow cytometry

T-DM1 was conjugated with the fluorescent dyes AlexaFluor488 or AlexaFluor647 (Thermo Fisher Scientific) according to the manufacturer's instructions.

To assess the competitive binding of T-DM1 with T-DXd or DV to HER2, N87 cells were first incubated with either unlabeled T-DXd (50 µg/mL) or unlabeled DV (50 µg/mL) for 30 min on ice. After washing, the cells were incubated with AlexaFluor488-labeled T-DM1 for an additional 30 min on ice. As a control, the cells were incubated only with AlexaFluor488-labeled T-DM1. After washing twice with phosphate-buffered saline (PBS) and fixation in 2 % formaldehyde, the cells were analyzed using a BD Accuri C6 Plus Flow Cytometer (Becton Dickinson, NJ, USA).

To study T-DM1 internalization, JIMT-1 cells were incubated at 37 °C for 15 or 30 min with either 50 µg/mL AlexaFluor647-labeled T-DM1 or a combination of 50 µg/mL DV and AlexaFluor647-labeled T-DM1. The samples were then treated with an acid strip buffer (0.5 M NaCl, 0.1 M glycine, pH 2.5) for 3 min on ice, washed, resuspended in 2 % formaldehyde, and analyzed using a BD Accuri C6 Plus Flow Cytometer. The internalized fraction of T-DM1 was calculated by dividing the mean fluorescence intensity of the acid-stripped sample by that of the non-acid-treated control [26]. Further detais are provided in the Supplement.

### Immunohistochemistry

Xenograft tumor samples were analyzed by immunohistochemistry using formalin-fixed, paraffin-embedded tissue as the starting material as previously described [19,21] and detailed in the Supplement. HER2 expression was assessed semi-quantitatively using a scale of negative (0), weakly positive (+), moderately positive (++), or strongly positive (+++), and quantitatively with the publicly available ImmunoMembrane HER2 IHC analysis web application, which generates scores for membrane staining intensity and completeness [27].

### Statistical analysis

In the xenograft tumor models, each treatment group consisted of five to six mice unless otherwise stated. Descriptive data are presented as the mean ± standard deviation. Groups were compared using one-way analysis of variance (ANOVA) or two-way repeated measures ANOVA. All treated animals were included in the survival analyses. Time to tumor progression was calculated using the Kaplan-Meier method, starting from the date of first treatment or from the date of tumor cell inoculation to the date of tumor progression or death, whichever occurred first. Overall survival was calculated from the date of first treatment or the date of inoculation to the date of death. Survival differences between groups were compared using the log-rank test. All *P* values are two-sided. Statistical analyses were carried out using IBM SPSS version 24 (IBM, Armonk, USA).

## Results

### Efficacy of single-agent T-DM1, T-DXd, and DV in breast and gastric cancer cell lines

T-DM1, T-DXd, and DV were not active in the HER2-negative breast cancer control cell line Hs-578T or in the HER2-positive gastric cancer lines SNU-216 and ROE19 in vitro. Among the remaining eight HER2-positive cell lines, DV exhibited at least comparable activity to T-DM1 and T-DXd, whereas T-DM1 had slightly greater activity than DV in the EFM-192A cell line. Additionally, DV inhibited the growth of RN87 gastric cancer cells, which were insensitive to T-DM1 and T-DXd (Table 1).

**Table 1:** In vitro efficacy of trastuzumab emtansine, trastuzumab deruxtecan, and disitamab vedotin.

**Cell line**	**Trastuzumab emtansine Mean IC_50_ (± SD)**^a^**µg/mL**	**Trastuzumab deruxtecan Mean IC_50_ (± SD)**^a^**µg/mL**	**Disitamab vedotin Mean IC_50_ (± SD)**^a^**µg/mL**
SKBR-3	0.017 (± 0.006)	0.159 (± 0.017)	0.007 (± 0.001)
UACC-812	0.033 (± 0.002)	0.046 (± 0.010)	0.012 (± 0.000)
EFM-192A	0.011 (± 0.003)	0.053 (± 0.011)	0.033 (± 0.002)
BT-474	0.036 (± 0.009)	0.090 (± 0.060)	0.033 (± 0.002)
JIMT-1	1.143 (± 0.243)	Not reached	0.050 (± 0.002)
OE19	0.249 (± 0.072)	Not reached	0.038 (± 0.001)
N87	0.132 (± 0.051)	0.106 (± 0.009)	0.028 (± 0.008)
SNU-216	Not reached	Not reached	Not reached
ROE19	Not reached	Not reached	Not reached
RN87	Not reached	Not reached	0.239 (± 0.031)
Hs-578T	Not reached	Not reached	Not reached

Abbreviations: HER2, human epidermal growth factor receptor 2; IC_50_, half-maximal (50 %) inhibitory concentration; SD, standard deviation.

a The mean of three experiments.

### Interactions between T-DM1, T-DXd, and DV in binding to HER2

**Fig. 1:**
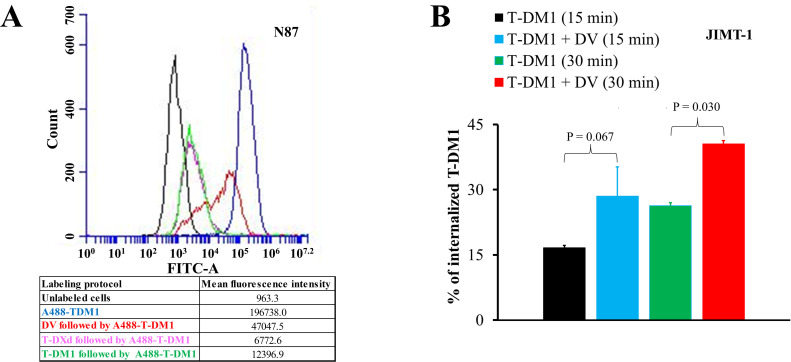
Interactions between T-DM1, T-DXd, and DV in binding to HER2. (A**)** Competitive binding of ADCs to HER2-positive N87 cancer cells was assessed with flow cytometry. 10,000 cells were measured per sample. (B**)** T-DM1 internalization was assessed with flow cytometry after incubating JIMT-1 cells with either AlexaFluor647-labeled T-DM1 (A647-T-DM1) or A647-T-DM1 plus DV for 15 and 30 min. Data shown are mean ± SD of three independent experiments. Means were compared using one-way ANOVA with Tukey's honestly significant difference post hoc test.

When HER2-positive N87 gastric cancer cells were preincubated with unlabeled T-DXd and subsequently with AlexaFluor488-labeled T-DM1, low binding of AlexaFluor488-labeled T-DM1 was observed. In contrast, when N87 cells were preincubated with unlabeled DV, AlexaFluor488-labeled T-DM1 still bound to the N87 cells, but there was less binding compared to when no preincubation was performed. This suggests that T-DM1 and DV partially compete for binding to HER2 (Fig. 1A).

DV increased T-DM1 internalization into JIMT-1 breast cancer cells. JIMT-1 cells internalized more AlexaFluor647-labeled T-DM1 when co-treated with DV compared to when AlexaFluor647-labeled T-DM1 was incubated alone, either for 15 min or 30 min (Fig. 1B).

### Activity of DV in combination with T-DM1 in breast and gastric cancer cell lines

Cells were then treated with increasing concentrations of T-DM1 or DV, both administered as single agents or in combination with the second ADC at a fixed concentration. The fixed concentrations of T-DM1 and DV used in the combinations were selected so that they exhibited only limited single-agent activity at these concentrations. T-DM1 plus a fixed dose of DV was more efficacious than T-DM1 alone in UACC-812, EFM-192A, and OE19 cells, but not in the HER2-negative Hs-578T cells (Supplementary Fig. S1). Additionally, DV plus a fixed concentration of T-DM1 resulted in improved activity compared to DV alone in all investigated HER2-positive cell lines (UACC-812, OE19, N87, and JIMT-1), but not in HER2-negative Hs-578T cells (Supplementary Fig. S1).

### DV as a single agent and in ADC combinations in breast and gastric cancer xenografts

**Fig. 2:**
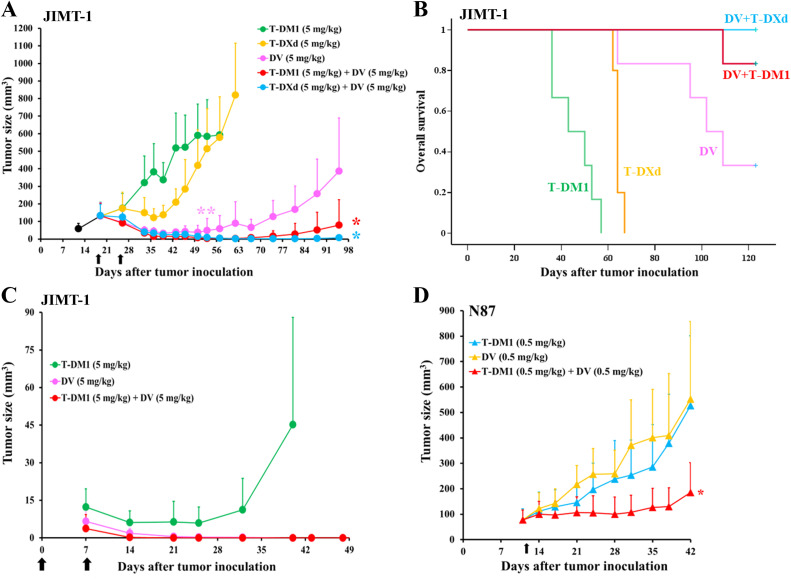
DV-containing ADC combinations are more effective than the single ADCs in breast and gastric cancer xenografts. (A**)** Mice bearing a JIMT-1 breast cancer xenograft were treated twice (black arrows) with either T-DM1, T-DXd, DV, T-DM1 plus concomitant DV, or T-DXd plus concomitant DV. Tumors treated with DV were smaller than the tumors in the T-DM1 (*P* = 0.003) or T-DXd (*P* = 0.004) groups (**, two-way repeated measures ANOVA with Tukey's honestly significant difference test). Tumors treated with T-DM1 plus DV or T-DXd plus DV were smaller than those in the DV treatment group (*, *P* = 0.023 and *P* = 0.011, respectively). (B**)** Overall survival since the day of tumor inoculation. Log-rank test, *P* < 0.001, comparing combination treatments to single-agent treatments. (C**)** Mice injected with JIMT-1 breast cancer cells were treated twice with either T-DM1 (5 mg/kg), DV (5 mg/kg), or T-DM1 plus concomitant DV (5 mg/kg of each) (black arrows). The first treatment was administered at the time of JIMT-1 cell inoculation (day 0). DV and T-DM1 plus concomitant DV eradicated all six tumors, whereas T-DM1 alone eradicated only one of the 17 tumors. (D**)** Mice bearing N87 gastric cancer xenografts were treated once (black arrow) with either T-DM1, DV, or T-DM1 plus concomitant DV. Tumors treated with T-DM1 plus DV (each 0.5 mg/kg) were smaller than those in the single-agent groups (*, *P* = 0.015, two-way repeated measures ANOVA). The mean (± SD) values are shown.

When mice with macroscopic JIMT-1 xenografts were treated with 5 mg/kg of DV, T-DM1, or T-DXd, tumors treated with T-DM1 continued to grow, the tumors treated with T-DXd shrank slightly before progressing, and tumors treated with DV responded (Fig. 2A). Anti-tumor efficacy was further improved when either 5 mg/kg T-DM1 or 5 mg/kg T-DXd was combined with 5 mg/kg DV, indicating that the DV plus T-DM1 and DV plus T-DXd combinations inhibited tumor growth more effectively than the corresponding single treatments (Fig. 2A). Mice in the combination groups survived longer than those in the single ADC groups (Fig. 2B), and tumors treated with the combinations progressed later than those treated with single ADCs (Supplementary Fig. S2A).

DV showed greater efficacy compared to T-DM1 in a JIMT-1 breast cancer xenograft model. In this model, treatment with T-DM1 (5 mg/kg), DV (5 mg/kg), or T-DM1 plus DV (each 5 mg/kg) was started at the time of JIMT-1 cell inoculation. Macroscopic tumors were observed in all treatment groups by day 7 after inoculation, and the tumors responded to the treatments later (Fig. 2C). Sixteen of the 17 tumors in the T-DM1-group began to grow around day 32 after inoculation (one mouse remained tumor-free until the end of the study), whereas in the DV and DV plus T-DM1 groups, the tumors disappeared in all treated mice. Tumors disappeared sooner in the T-DM plus DV group compared to the DV group (Supplementary Fig. S3).

Single-agent T-DM1 and DV showed substantial and similar activity for N87 gastric cancer xenografts at a dose of 5 mg/kg (Supplementary Fig. S4). Therefore, the mice were treated with a lower ADC dose (0.5 mg/kg) to evaluate the efficacy of the drug combination. The T-DM1 plus DV combination was more effective than the single ADCs in this model. N87 tumors treated with 0.5 mg/kg T-DM1 plus 0.5 mg/kg DV were smaller than those treated with either 0.5 mg/kg T-DM1 or 0.5 mg/kg DV (Fig. 2D), and the mice treated with 0.5 mg/kg T-DM1 plus 0.5 mg/kg DV also survived longer than those that received the single-agent treatments (Supplementary Fig. S5).

The combination therapies were well tolerated, and the weight of the mice treated with an ADC combination was similar to that of mice treated with a single agent (Supplementary Fig. S6A-C).

In summary, DV demonstrated substantial antitumor activity in both breast cancer and gastric cancer xenograft models, and its efficacy was further enhanced when co-administered with either T-DM1 or T-DXd.

### DV inhibits breast and gastric cancer xenografts that progress after T-DM1 and T-DXd treatment

**Fig. 3:**
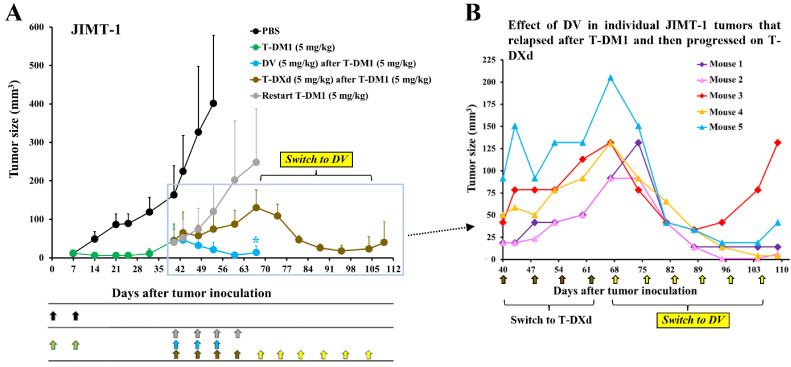
DV inhibits breast cancer xenografts that progress after T-DM1 and T-DXd treatment. (A**)** Mice injected with JIMT-1 breast cancer cells were treated twice with either PBS or T-DM1 on day 0 and day 7 (black and green arrows, respectively). Tumors that relapsed after T-DM1 were treated with either DV (blue arrows), T-DXd (brown arrows), or further T-DM1 (grey arrows) from day 40 onwards. Tumors treated with DV after T-DM1 were smaller than those in the T-DXd after T-DM1 group (*, *P* = 0.005, two-way repeated measures ANOVA). Treatment of mice in the T-DXd after T-DM1 group was switched to DV (yellow arrows; from day 68 onwards), which resulted in tumor shrinkage. (B**)** Details of the JIMT-1 tumors that relapsed after T-DM1 treatment and progressed on subsequent T-DXd treatment (the brown boxed curve in panel A). The tumors responded when T-DXd treatment (5 mg/kg, brown arrows) was switched to DV (yellow arrows, 5 mg/kg). The mean (± SD) values are shown.

In a JIMT-1 breast cancer model described above (Fig. 3A), 16 of the 17 mice treated with T-DM1 had progressed by day 40. Of these, five tumors were treated with DV, five with T-DXd, and five with T-DM1, while the remaining mouse, which had a large tumor, was euthanized. Tumors treated with T-DM1 or T-DXd progressed, whereas all tumors treated with DV responded. We then switched the treatment of the five tumors that had progressed on T-DXd from T-DXd to DV, and all five tumors responded. Three of these five tumors remained small until the end of the study, while two progressed (Fig. 3A,B).

**Fig. 4:**
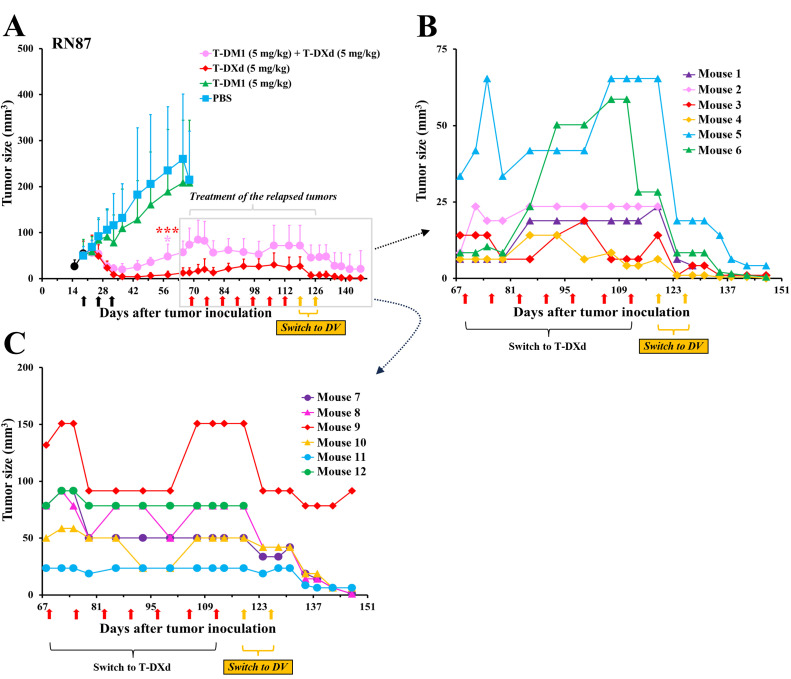
DV inhibits gastric cancer xenografts that progress after T-DM1 and T-DXd treatment. **(**A**)** Mice with RN87 gastric cancer xenografts were treated three times (black arrows) with either PBS, T-DM1, T-DXd, or T-DM1 plus T-DXd. Relapsed tumors (boxed) were first treated with seven doses of T-DXd (red arrows, 5 mg/kg) and later with two doses of DV (orange arrows, 5 mg/kg). Tumors treated with T-DM1 plus T-DXd were smaller than those in the T-DM1 group (*, *P* = 0.032), but larger than those in the T-DXd group (***, *P* = 0.0005, two-way repeated measures ANOVA). (B**), (**C**)** Detailed presentation of the individual RN87 tumors that relapsed after T-DXd (B) or relapsed after T-DM1 plus T-DXd (C). All relapsed RN87 tumors responded when the treatment was switched to DV. The mean (± SD) values are shown.

Both T-DXd and the T-DM1 plus T-DXd combination showed substantial efficacy against RN87 gastric cancer tumors, but neither treatment eradicated the tumors (Fig. 4A). A second course of seven T-DXd injections stabilized five of the six tumors that had progressed after prior T-DXd treatment. However, when the treatment was switched to DV, all five tumors responded (one mouse was sacrificed for histology sampling before starting DV) (Fig. 4B). Similarly, a course of T-DXd stabilized the RN87 tumors that had progressed after prior T-DM1 plus T-DXd treatment, but when the treatment was switched to DV, all tumors shrank (Fig. 4C). By the end of the study, eight of the nine DV-treated tumors were small, and only one was progressing (Fig. 4B,C).

In these models, DV was effective in treating breast cancer and gastric cancer xenograft tumors that were resistant to T-DM1, T-DXd, or their combination.

### Efficacy of T-DXd in T-DM1-resistant gastric cancer RN87 xenograft tumors

The RN87 gastric cancer cell line was insensitive to T-DM1 and T-DXd in vitro (Table 1). In line with this, the RN87 xenograft tumors treated with T-DM1 progressed, while they responded rapidly to T-DXd (Fig. 4A). When the T-DXd-treated tumors began to re-grow, subsequent T-DXd treatments stabilized five of the six tumors (Fig. 4B). The activity of the T-DM1 plus T-DXd combination was lower than that of T-DXd alone in the RN87 xenografts (Fig. 4A). The body weight of the mice treated with the T-DM1 plus T-DXd combination remained similar to that of mice in the single-agent treatment groups (Supplementary Fig. S6D).

### Effect of ADC treatments on HER2 expression

**Fig. 5:**
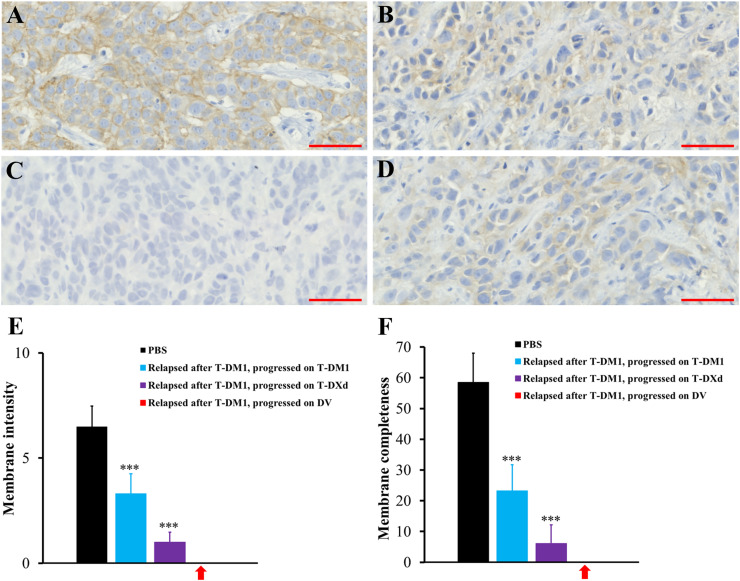
JIMT-1 breast cancer xenograft HER2 expression was assessed using immunohistochemistry. (A**)** A tumor treated with PBS. HER2 expression was graded as ++ on a scale from 0 (no expression), + (weak), ++ (moderate), to +++ (high expression). (B**)** A tumor that relapsed after T-DM1 treatment and subsequently progressed on T-DM1 treatment (+). (C**)** A tumor that relapsed after T-DM1 treatment and subsequently progressed on DV treatment (0). (D**)** A tumor that relapsed after T-DM1 treatment and subsequently progressed on T-DXd treatment (+). In panels A-D, the scale bar = 50 µm. (E), (F) Cell membrane HER2 expression staining intensity (E) and completeness (F) were quantified using the ImmunoMembrane HER2 immunohistochemistry analysis software. The red arrows point to tumors that relapsed after T-DM1 treatment, subsequently progressed on DV, and showed no detectable HER2 expression. The mean ± SD values from ten randomly selected representative tumor fields are shown. ***, P < 0.001 compared to the PBS group. One-way ANOVA with Tukey's honestly significant difference test was used to compare the means.

JIMT-1 breast cancer xenografts treated with T-DM1 or T-DXd typically expressed less HER2 in immunohistochemistry (usually 1+ on a scale from 0 to 3+) compared to JIMT-1 xenografts treated with PBS (typically 2+; Fig. 5). The tumors that relapsed after T-DM1 treatment and that subsequently responded to DV but later progressed (as shown in the model in Fig. 3B) had lost HER2 expression (Fig. 5C,E,F). In contrast, RN87 gastric cancer xenografts that progressed on T-DM1, T-DXd, or both, or progressed on DV (as shown in the model in Fig. 4A), still expressed HER2 strongly (Supplementary Fig. S7A-E). Giant multinucleated cells were present in RN87 tumors that had first responded to T-DXd but subsequently progressed, and then either stabilized or progressed on subsequent T-DXd treatment (as shown in the model in Fig. 4A) (Supplementary Fig. S7C).

## Discussion

T-DM1 and T-DXd are recommended treatments for both early and advanced HER2-positive breast cancer, as well as advanced gastric cancer [28]. However, most patients treated eventually progress [8,12,14], and such patients have limited therapeutic options. In this study, we evaluated the efficacy of DV, T-DM1, and T-DXd both as single agents and in combinations across several experimental models of HER2-positive breast and gastric cancer. We found that DV was effective in both breast and gastric cancer cell lines, as well as in mouse xenograft tumors resistant to T-DM1, T-DXd, or both. Moreover, combinations of DV with T-DM1 and DV with T-DXd were more effective than the corresponding single ADCs (Supplementary Fig. S8).

The three ADCs investigated bind to HER2, but they are highly distinct constructs. Hertuzumab, the antibody backbone of DV, has a higher affinity for HER2 than trastuzumab [18], which is the antibody backbone of both T-DM1 and T-DXd. Although both trastuzumab and hertuzumab evoke antibody-dependent cellular cytotoxicity (ADCC), which is preserved in T-DM1, T-DXd, and DV [3,18,20,29], hertuzumab induces ADCC more potently than trastuzumab [18]. DV and T-DM1 deliver a microtubule assembly inhibitor payload (MMAE and DM1, respectively) to cancer cells, whereas T-DXd carries the topoisomerase I inhibitor deruxtecan [5,13,18]. Both deruxtecan and MMAE are membrane-permeable and can enter neighboring cells, causing bystander cell death. In contrast, T-DM1 lacks this bystander effect because its active metabolite (lysine-MCC-DM1) is not cell membrane-permeable [5,30,31].

The data from our xenograft models suggest that DV is effective against HER2-positive cancers that progress on T-DM1 and/or T-DXd or stabilize during T-DXd treatment. HER2 was downregulated but still detectable in JIMT-1 breast cancer xenografts (immunohistochemistry score 1+) that had progressed on both T-DM1 and T-DXd, suggesting that these cross-resistant tumors remain potentially targetable with anti-HER2 therapies. Indeed, DV was highly effective in these tumors. Our findings are consistent with observations from a recent retrospective clinical study, which suggests that DV is effective following trastuzumab-based ADC therapy in breast cancer [32].

A few JIMT-1 tumors that eventually progressed even during subsequent DV treatment were found to be HER2-negative by immunohistochemistry. Loss of HER2 expression has also been linked with resistance to next-generation anti-HER2 ADCs, such as ARX788 and XMT-1522, in breast cancer xenografts [21,22]. HER2 was not downregulated in RN87 gastric cancer xenograft tumors that were cross-resistant to T-DM1 and T-DXd, and these tumors responded to subsequent DV treatment. In addition to HER2 downregulation, the expression of ABC transporters or increased expression of the human epidermal growth factor receptor may partially explain drug resistance in the current models [21,22,33,34]. RN87 cells strongly express the multidrug resistance-inducing drug transporters ABCC1, ABCC2, and ABCG2 [21,22]. Nevertheless, these cells were sensitive to DV, suggesting that MMAE may be a less avid substrate for these transporters compared to lysine-MCC-DM1, the active metabolite of T-DM1, or deruxtecan, the payload of T-DXd.

Other suggested molecular mechanisms that may contribute to resistance to T-DM1 or T-DXd in cancer include the loss of solute carrier family 46 member 3 (SLC46A3), phosphatase and tensin homolog (PTEN) deficiency, caveolin 1 (CAV1) overexpression, multidrug resistance protein 1 (MDR1) expression, depletion of SLX4 structure-specific endonuclease subunit, and overexpression of *voltage-dependent anion channel 3* (*VDAC3*)-derived circular RNA [[35], [36], [37], [38]]. Whether these mechanisms influence the efficacy of DV in the treatment of T-DM1- or T-DXd-resistant breast or gastric cancers warrants further investigation.

The combinations of T-DM1 plus DV and T-DXd plus DV had a stronger anti-tumor effect than the respective single ADCs, whereas the combination of T-DM1 plus T-DXd did not demonstrate enhanced efficacy. In fact, the T-DM1 plus T-DXd combination was less effective in RN87 gastric cancer xenografts than T-DXd alone, likely due to T-DM1 interfering with the binding of T-DXd, which was the more effective drug in this model. Combining ADCs with different antibody backbones may provide a more rational approach. Indeed, co-treatment with T-DM1 plus DV resulted in a greater efficacy compared to the respective single ADCs in several breast and gastric cancer cell lines (EFM-192A, UACC-812, JIMT-1, OE19, and N87) in vitro. Additionally, mice treated with T-DM1 plus DV had smaller N87 gastric cancer xenografts than those treated with the single ADCs. Similarly, mice with JIMT-1 breast cancer xenografts treated with either T-DM1 plus DV or T-DXd plus DV had smaller tumors and survived longer than those treated with the single ADCs. Furthermore, in another JIMT-1 breast cancer model, where treatments were administered starting from the day of tumor inoculation, the T-DM1 plus DV combination eradicated the tumors more rapidly than DV alone, while the tumors in the T-DM1-only group progressed. To our knowledge, this is the first study to report that the combination of two anti-HER2 ADCs may have greater anti-tumor efficacy than the corresponding single-agent ADCs.

A combination of antibodies targeting two non-overlapping epitopes on HER2 may induce receptor cross-linking and clustering, which triggers rapid internalization [39,40]. In agreement with these observations, we found that co-treatment with T-DM1 and DV increased T-DM1 internalization, despite T-DM1 and DV interact upon binding to HER2. Therefore, co-administration of DV with T-DM1 or T-DXd could enhance efficacy in two ways: (1) by exposing cancer cells to two cytotoxic payloads instead of one, mimicking combination chemotherapy, and (2) by inducing rapid ADC internalization, leading to faster accumulation of cytotoxic payloads within the cancer cells. Both DM1 and MMAE are microtubule assembly inhibitors, but they bind to different sites on tubulin and inhibit microtubule polymerization in distinct ways [[41], [42], [43]], while deruxtecan is a topoisomerase I inhibitor [13]. A cocktail of anti-HER2 ADCs with distinct binding sites on HER2 and carrying different payloads could further improve efficacy. Some of the anti-HER2 ADCs currently being developed harbor antibodies that bind to epitopes different from trastuzumab, suggesting they may be well-suited for inclusion in an ADC cocktail [18,22,44]. Novel anti-HER2 ADCs with structural innovations are being developed [37,45], and combinations of anti-HER2 ADCs with other types of anti-cancer agents such as immune checkpoint inhibitors, tyrosine kinase inhibitors, or CDK4/6 inhibitors are also being investigated [37,46].

The safety profile of DV suggests that its co-administration with T-DXd or T-DM1 is feasible, but this remains to be confirmed in clinical trials. In a phase I trial evaluating DV, the most frequently observed grade 3 or higher adverse events were neutropenia (18 % of patients), elevated blood gamma-glutamyl transferase concentration (13 %), and asthenia (11 %). Grade 1 or 2 peripheral neuropathy, an off-target effect of the MMAE payload, was common, but no grade 3 or higher neuropathy occurred in those patients who received 10 mg of intravenous dexamethasone prior to each DV infusion. Interstitial lung disease and cardiac toxicity were infrequent [47].

A randomized clinical trial demonstrated superior efficacy of T-DXd compared to T-DM1 in patients with advanced breast cancer [13], and T-DXd has proven effective in treating breast cancer patients who progress on T-DM1 [14]. Consistent with these findings, we observed that T-DXd was more active than T-DM1 in JIMT-1 breast cancer xenografts and inhibited the growth of T-DM1-resistant JIMT-1 breast and RN87 gastric cancer xenografts.

We observed giant multinucleated cancer cells in RN87 xenografts treated with T-DXd. These cells are a hallmark of mitotic catastrophe and have also been observed in breast and gastric cancers treated with T-DM1 or XMT-1522 [20,22,24]. To our knowledge, this is the first study to report that T-DXd treatment may lead to the formation of giant multinucleated cancer cells. Topoisomerase I inhibitors can induce DNA damage and cell cycle arrest [48,49], which may lead to mitotic catastrophe [50] and could explain the formation of giant multinucleated cancer cells induced by T-DXd.

The study has some limitations. The contribution of the adaptive immune system to ADC efficacy could not be assessed due to the immune deficiencies in the SCID mice. We did not conduct toxicity studies, but we observed no weight loss or deterioration in the body condition score in the mice treated with the single ADCs or their combinations.

## Conclusion

The findings support investigating the efficacy of DV in patients with HER2-positive breast or gastric cancer who have progressed during or after treatment with T-DM1 or T-DXd. Additionally, the data suggest that administering DV in combination with T-DM1 or T-DXd may result in superior efficacy compared to using the single ADCs alone.

## Ethics approval

The Committee for animal experiments of the District of Southern Finland approved the animal experiments under licenses ESAVI/403/2019, ESAVI/11,614/2022, and ESAVI/10,262/2022. Animal studies were conducted in accordance with Guidance on the Operation of the Animals (Scientific Procedures) Act 1986 and associated guidelines, EU Directive 2010/63 on the protection of animals used for scientific purposes. The reporting in the manuscript follows the recommendations in the ARRIVE guidelines (https://arriveguidelines.org/arrive-guidelines).

## Consent for publication

Not applicable.

## Availability of data and materials

All data supporting the findings of this study are included within the manuscript and the supplementary figures. The raw data used for analysis are available from the corresponding author upon a request.

## Funding

The study was supported by grants from the Sigrid Jusélius Foundation (H.J.), the Jane and Aatos Erkko Foundation (H.J.), and the Cancer Society of Finland (H.J.).

## CRediT authorship contribution statement

**Negar Pourjamal:** Writing – review & editing, Visualization, Validation, Methodology, Investigation, Formal analysis, Data curation. **Vadim Le Joncour:** Writing – review & editing, Methodology, Investigation, Data curation. **György Vereb:** Writing – review & editing, Resources, Project administration, Methodology. **Cilla Honkamaki:** Methodology, Investigation, Data curation. **Jorma Isola:** Writing – review & editing, Software, Resources, Project administration, Methodology. **Jeffrey V Leyton:** Writing – review & editing, Resources, Project administration, Methodology. **Pirjo Laakkonen:** Writing – review & editing, Supervision, Resources, Project administration. **Heikki Joensuu:** Writing – review & editing, Writing – original draft, Supervision, Resources, Project administration, Funding acquisition. **Mark Barok:** Writing – review & editing, Writing – original draft, Visualization, Validation, Supervision, Resources, Project administration, Methodology, Investigation, Formal analysis, Data curation, Conceptualization.

## Declaration of competing interest

J.I. is the owner of Jilab Inc. J.V.L. is listed on patents unrelated to the research in this work, holds options from Defence Therapeutics Inc., and has consulted for PinotBio and Merck within the past 5-years. H.J. is the Chair of the Scientific Advisory Boards of Orion Pharma, Neutron Therapeutics, and Maud Kuistila Foundation, from which work he has received financial compensation, and owns stock in Orion Pharma and Sartar Therapeutics. M.B. has an advisory relationship at ProBiont Ltd. M.B. and H.J. report grants from Defense Therapeutics Inc. and Merck KGaA outside the submitted work. No potential conflicts of interest were disclosed by the other authors.

## Appendix Supplementary materials

Image, application 1

Image, image 2

Image, image 3

Image, image 4

Image, image 5

Image, image 6

Image, image 7

Image, image 8

Image, image 9
