# Supplementary material for: Disitamab vedotin in preclinical models of HER2-positive breast and gastric cancers resistant to trastuzumab emtansine and trastuzumab deruxtecan
Source: Transl Oncol. 2025 Jan 20;53:102284. doi: 10.1016/j.tranon.2025.102284 (PMC11788861; doi:10.1016/j.tranon.2025.102284)
Supplement: Supplementary file 1 [file mmc1.docx]

**Supplement**

**Contents**

Supplementary methods p. 2

Cell viability assay p. 2

Immunohistochemistry p. 2

Gating used in flow cytometry p. 3

Supplementary Table S1 p. 4

Supplementary Table S2 p. 5

References p. 6

Legends to the Supplementary Figs. 1-8 p. 7

**Supplementary methods**

*Cell viability assay*

The effects of T-DM1 (Roche Ltd., Basel, Switzerland), T-DXd (AstraZeneca, Cambridge, UK), and DV (MedChemExpress, Monmouth Junction, NJ, USA) on cell growth were studied using the AlamarBlue method (Thermo Fisher Scientific, Waltham, USA) (1, 2) . Briefly, the cells were trypsinized and plated in 96-well flat-bottomed tissue culture plates. The efficacy of each ADC was first tested as a single agent at concentrations of 0.0001, 0.0006, 0.003, 0.016, 0.08, 0.4, 1, 2, and 10 µg/mL. Next, two ADC combinations were investigated: (1) increasing concentrations of T-DM1 with a fixed concentration of DV, and (2) increasing concentrations of DV with a fixed concentration of T-DM1. In these experiments, the increasing concentrations of T-DM1 or DV were 0.0001, 0.0006, 0.003, 0.016, 0.08, 0.4, 1, and 2 µg/mL. The fixed ADC concentration was chosen so that the ADC, as a single agent, had no or only minor inhibitory effect on cell growth. After five days of incubation, 10 µL of the AlamarBlue reagent (Thermo Fisher Scientific) was added to the culture medium, and the number of viable cells was assessed by measuring fluorescence after excitation at 540 nm and emission at 590 nm using a PHERAstar FS plate reader (BMG Labtech, Ortenberg, Germany). Sample fluorescence was normalized to the fluorescence of the cell-free culture medium. The proportion of viable cells was obtained by dividing the test sample fluorescence by a PBS-treated control sample fluorescence. The drug dose that achieved the half maximal (50%) inhibitory concentration (IC_50_) was calculated using a GraphPad Prism software (GraphPad Software, San Diego, USA) using a four-parameter nonlinear regression model.

*Immunohistochemistry*

Xenograft tumor samples were stained by immunohistochemistry as previously described (1, 3). Tissue samples from xenograft tumors were fixed in 4% buffered formaldehyde for 24 hours, processed into paraffin, and sectioned. For immunohistochemistry, 4 μm sections were deparaffinized and subjected to antigen-retrieval in a sodium citrate buffer (10 mM Sodium Citrate, pH 6.0) using a 2100 Antigen Retriever (Aptum Biologics Ltd., Southampton, UK), following the manufacturer's recommendations. After blocking the non-specific binding, the anti-HER2 primary antibody (SP3, Thermo Fisher Scientific, Waltham, MA, USA) was applied at optimized concentration, and incubated overnight at 4 °C. The primary antibody binding was detected using a BrightVision Poly-HRP anti mouse kit (VWR, Radnor, USA) and 3,3′-diaminobenzidine (ImmPACT DAB, Vector Laboratories, Burlingame, CA, USA) following the manufacturer's recommendations. The tissue sections were counterstained with hematoxylin. The stained slides were imaged using an 20x objective of an Olympus BX50 microscope (Olympus Corporation, Tokyo, Japan) integrated with a SlideStrider objective slide scanner (JILab Inc., Tampere, Finland) or with a 20x objective on a Zeiss Axio Scan.Z1 Slide Scanner (Carl Zeiss, Göttingen, Germany). HER2 expression was visualized and quantified semi-quantitatively using a scale of negative (0), weakly positive (+), moderately positive (++), or strongly positive (+++), and quantitatively using the publicly available ImmunoMembrane HER2 IHC analysis web application, which generates scores for membrane staining intensity and completeness (4). The data are presented as the average membrane staining intensity and completeness scores (± standard deviation), both assessed from a minimum of ten randomly selected representative fields.

*Gating used in flow cytometry*

Forward scatter and side scatter were used to create gates that excluded debris and cell clumps. The fluorescence intensity of the events (cells) within the gate was then measured. No other types of gates were applied.

**Supplementary Table S1.**

HER2 targeting antibody-drug conjugates studied.

| ADC name | Monoclonal antibody | Linker | Payload | DAR | Payload action | Bystander killing effect (ref) |
| --- | --- | --- | --- | --- | --- | --- |
| Trastuzumab emtansine  (T-DM1, Kadcyla^®^) | Trastuzumab | Non-cleavable  SMCC | DM1;  maytansine derivative | 3.5 | Microtubule inhibitor | No (5) |
| Trastuzumab deruxtecan  (T-DXd, Enhertu^®^, DS-8201a) | Trastuzumab | Cleavable  µGGFG | Deruxtecan; exatecan derivative | 8 | Topoisomerase I inhibitor | Yes (5) |
| Disitamab vedotin (DV, Aidixi^®^, RC48-ADC, hertuzumab-vc-MMAE) | Hertuzumab | Cleavable  MC-VC-PABC | Monomethyl auristatin E | 4 | Microtubule inhibitor | Yes (6) |

Abbreviations: ADC, antibody-drug conjugate; DAR, drug-antibody ratio; MC-VC-PABC, cathepsin cleavable maleimidocaproyl-valine-citrulline-p-aminobenzylalcohol-p-nitrophenyl carbonate; µGGFG, enzymatically cleavable maleimide glycine-glicyne-phenylalanine-glycine; SMCC, N-succinimidyl-4-(N-maleimidomethyl) cyclohexane-1-carboxylate.

**Supplementary Table S2.**

The cell lines studied.

| **Human cell line** | **HER2 expression** | **Source** | **Reported sensitivity to T-DM1 (ref.)** |
| --- | --- | --- | --- |
| *Breast cancer* |  |  |  |
| BT-474 | Positive | ATCC | Sensitive (7, 8) |
| EFM-192A | Positive | DSMZ | Sensitive (7, 8) |
| JIMT-1 | Positive | Laboratory of Cancer Biology, University of Tampere, Finland^*^ | Sensitive (7, 8) |
| SKBR-3 | Positive | ATCC | Sensitive (7, 8) |
| UACC-812 | Positive | ATCC | Sensitive (7, 8) |
| Hs-578T | Negative | ATCC | Resistant (1) |
| *Gastric cancer* |  |  |  |
| N87 | Positive | ATCC | Sensitive (9) |
| RN87 | Positive | Established in our laboratory from N87 | Resistant (1, 2) |
| OE19 | Positive | ECCC | Sensitive (9) |
| ROE19 | Positive | Established in our laboratory from OE19 | Resistant (1, 2) |
| SNU-216 | Positive | KCLB | Resistant (2, 9) |

Abbreviations: ATCC, American Type Tissue Culture Collection (Manassas, VA, USA); DSMZ, German Resource Center for Biological Material, Leibniz Institute (Braunschweig, Germany); ECCC, European Collection of Cell Culture (Salisbury, United Kingdom); HER2, human epidermal growth factor receptor 2; KCLB, Korean Cell Line Bank (Seoul, Republic of Korea).

^*^The JIMT-1 cell line is available also from DSMZ.

**References**

1. Barok M, Le Joncour V, Martins A, Isola J, Salmikangas M, Laakkonen P, et al. ARX788, a novel anti-HER2 antibody-drug conjugate, shows anti-tumor effects in preclinical models of trastuzumab emtansine-resistant HER2-positive breast cancer and gastric cancer. Cancer Lett. 2020;473:156-63.

2. Le Joncour V, Martins A, Puhka M, Isola J, Salmikangas M, Laakkonen P, et al. A Novel Anti-HER2 Antibody-Drug Conjugate XMT-1522 for HER2-Positive Breast and Gastric Cancers Resistant to Trastuzumab Emtansine. Mol Cancer Ther. 2019;18(10):1721-30.

3. Pourjamal N, Yazdi N, Halme A, Joncour VL, Laakkonen P, Saharinen P, et al. Comparison of trastuzumab emtansine, trastuzumab deruxtecan, and disitamab vedotin in a multiresistant HER2-positive breast cancer lung metastasis model. Clin Exp Metastasis. 2024;41(2):91-102.

4. Tuominen VJ, Tolonen TT, Isola J. ImmunoMembrane: a publicly available web application for digital image analysis of HER2 immunohistochemistry. Histopathology. 2012;60(5):758-67.

5. Ogitani Y, Hagihara K, Oitate M, Naito H, Agatsuma T. Bystander killing effect of DS-8201a, a novel anti-human epidermal growth factor receptor 2 antibody-drug conjugate, in tumors with human epidermal growth factor receptor 2 heterogeneity. Cancer Sci. 2016;107(7):1039-46.

6. Shi F, Liu Y, Zhou X, Shen P, Xue R, Zhang M. Disitamab vedotin: a novel antibody-drug conjugates for cancer therapy. Drug Deliv. 2022;29(1):1335-44.

7. Barok M, Tanner M, Koninki K, Isola J. Trastuzumab-DM1 causes tumour growth inhibition by mitotic catastrophe in trastuzumab-resistant breast cancer cells in vivo. Breast Cancer Res. 2011;13(2):R46.

8. Koninki K, Barok M, Tanner M, Staff S, Pitkanen J, Hemmila P, et al. Multiple molecular mechanisms underlying trastuzumab and lapatinib resistance in JIMT-1 breast cancer cells. Cancer Lett. 2010;294(2):211-9.

9. Barok M, Tanner M, Koninki K, Isola J. Trastuzumab-DM1 is highly effective in preclinical models of HER2-positive gastric cancer. Cancer Lett. 2011;306(2):171-9.

**Legends to the supplementary figures**

**Supplementary Fig. S1.** Effects of DV, T-DM1, and their combinations on breast and gastric cancer cell lines. (A-F) Cells were treated with increasing concentrations of either DV or T-DM1, or with increasing concentrations of T-DM1 plus a fixed concentration of DV, or with increasing concentrations of DV plus a fixed concentration of T-DM1. Means ± SD from at least three independent experiments were plotted, with each experiment containing four replicates. Means were compared using a two-way repeated measures ANOVA with Tukey's honest significant difference post hoc test. *, P ˂ 0.05; **, P ˂ 0.01; ***, P ˂ 0.001.

**Supplementary Fig. S2.** Progression-free survival of mice injected with JIMT-1 breast cancer cells and treated with either T-DM1 (5 mg/kg), T-DXd (5 mg/kg), DV (5 mg/kg), DV plus concomitant T-DM1 (5 mg/kg of each), or DV plus concomitant T-DXd (5 mg/kg of each). The first treatment was administered at the time of JIMT-1 cell inoculation (day 0). Log-rank test P < 0.001 between the combination treatments and single-agent treatments.

**Supplementary Fig. S3.** The proportion of mice with palpable JIMT-1 tumor in the experiment shown in Figure 2C. Mice injected with JIMT-1 breast cancer cells were treated twice with either T-DM1 (5 mg/kg), DV (5 mg/kg), or T-DM1 plus concomitant DV (5 mg/kg of each). The first treatment was administered at the time of JIMT-1 cell inoculation (day 0). DV and T-DM1 plus DV treatments eradicated all six tumors, whereas T-DM1 eradicated one of 17 tumors. Tumors detected on day 7 and treated with T-DM1 plus DV disappeared earlier than those treated with the single ADCs. Time with tumor was calculated from the date of tumor inoculation. Log-rank test P < 0.01.

**Supplementary Fig. S4.** Effects of DV and T-DM1 on N87 gastric cancer xenografts. Mice bearing N87 gastric cancer xenografts were treated once (black arrow) with either T-DM1 (5 mg/kg) or DV (5 mg/kg). Mean (± SD) values are shown.

**Supplementary Fig. S5.** Overall survival from the day of tumor inoculation of mice with N87 gastric cancer xenografts treated with either T-DM1, DV, or T-DM1 plus concomitant DV. Mice were treated with either T-DM1 (0.5 mg/kg), DV (0.5 mg/kg), or T-DM1 plus DV (0.5 mg/kg of each) on day 12 (experiment shown in Fig. 2D). Mice treated with T-DM1 plus DV survived longer than those that received the single ADCs. Log-rank test P < 0.01 between the combination treatment and single-agent treatments.

**Supplementary Fig. S6.** Weight of mice treated with anti-HER2 ADCs and their combinations. (A) Mice bearing a JIMT-1 breast cancer xenograft treated with either T-DM1, T-DXd, DV, T-DM1 plus concomitant DV, or T-DXd plus concomitant DV. (B) Mice with a JIMT-1 breast cancer xenograft treated with either PBS, T-DM1, DV, or with T-DM1 plus concomitant DV on days 0 and 7 (arrows). Mice with relapsed tumors after T-DM1 treatment were subsequently treated with DV (blue arrows), T-DXd (brown arrows), or additional T-DM1 (grey arrows) starting from the day 40. The T-DXd group was switched to DV (yellow arrows, 5 mg/kg) on day 68. (C) Mice with N87 gastric cancer xenografts were treated with either T-DM1, DV, T-DM1 plus concomitant DV. (D) Mice with RN87 gastric cancer xenografts were treated with either PBS, T-DM1, T-DXd, or T-DM1 plus concomitant T-DXd. The orange arrowheads in panels A, C, and D indicate the days of drug administration. Group mean (± SD) weights are plotted from the date of tumor inoculation over the duration of the study.

**Supplementary Fig. S7.** RN87 gastric cancer xenografts retained HER2 expression after the treatments. (A) HER2 expression of a tumor treated with PBS was graded +++ in immunohistochemistry. (B) A tumor that progressed on T-DM1 (+++). (C) A tumor that relapsed after T-DXd treatment and then progressed on subsequent T-DXd treatment (+++). (D) A tumor that relapsed after T-DM1 plus concomitant T-DXd treatment and then progressed on subsequent T-DXd (+++). (E) A tumor that relapsed after T-DM1 plus concomitant T-DXd treatment and then progressed on subsequent T-DXd and later on subsequent DV. The black arrows in (C) point to giant multinucleated cancer cells. The bar = 50 µm.

**Supplementary Fig. S8.** Graphical abstract of the study.
