# Supplementary figures and images for: Disitamab vedotin in preclinical models of HER2-positive breast and gastric cancers resistant to trastuzumab emtansine and trastuzumab deruxtecan
Source: Transl Oncol. 2025 Jan 20;53:102284. doi: 10.1016/j.tranon.2025.102284 (PMC11788861; doi:10.1016/j.tranon.2025.102284)

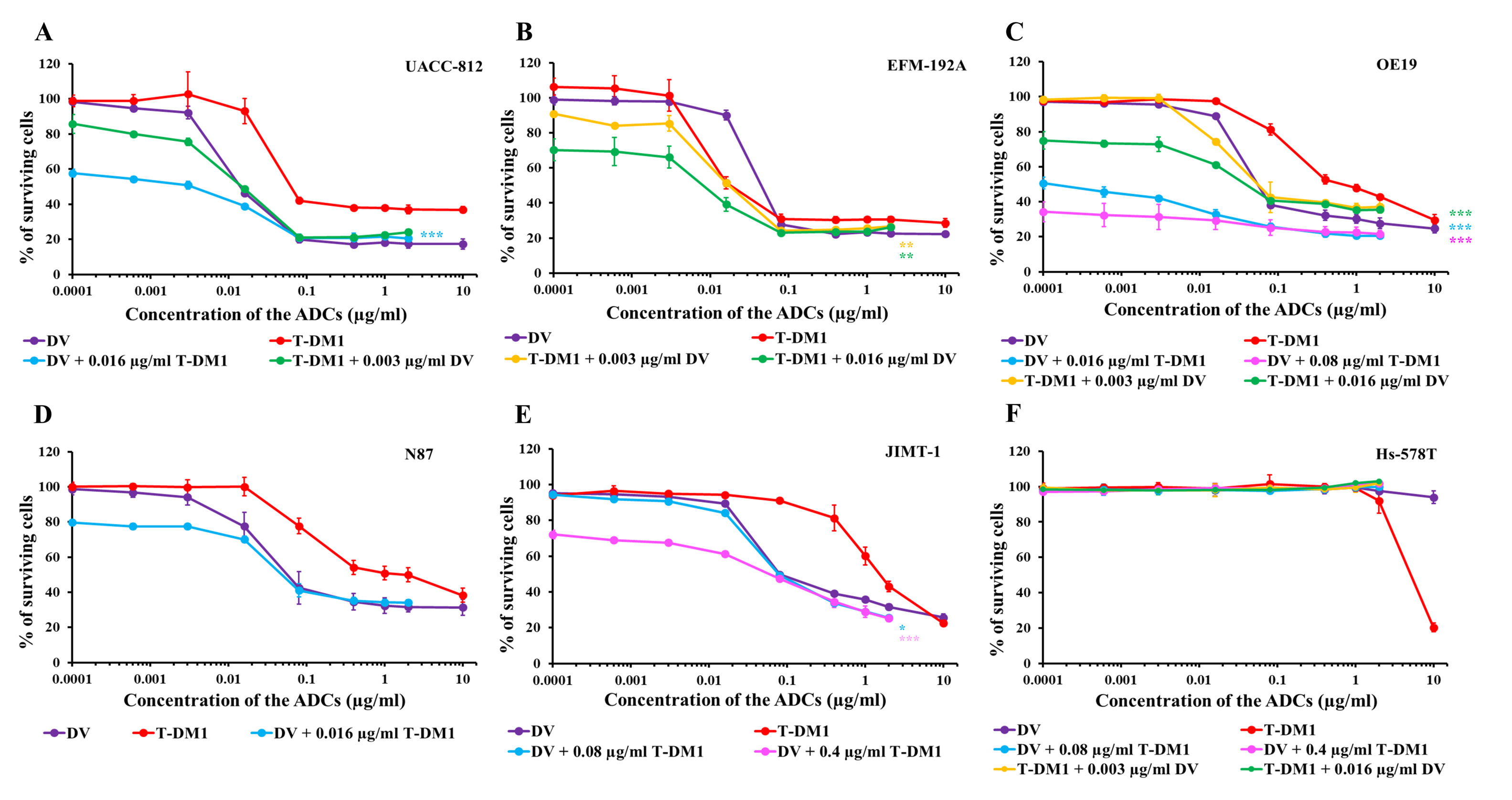

Supplement: Supplementary file 2 [file mmc2.jpg]

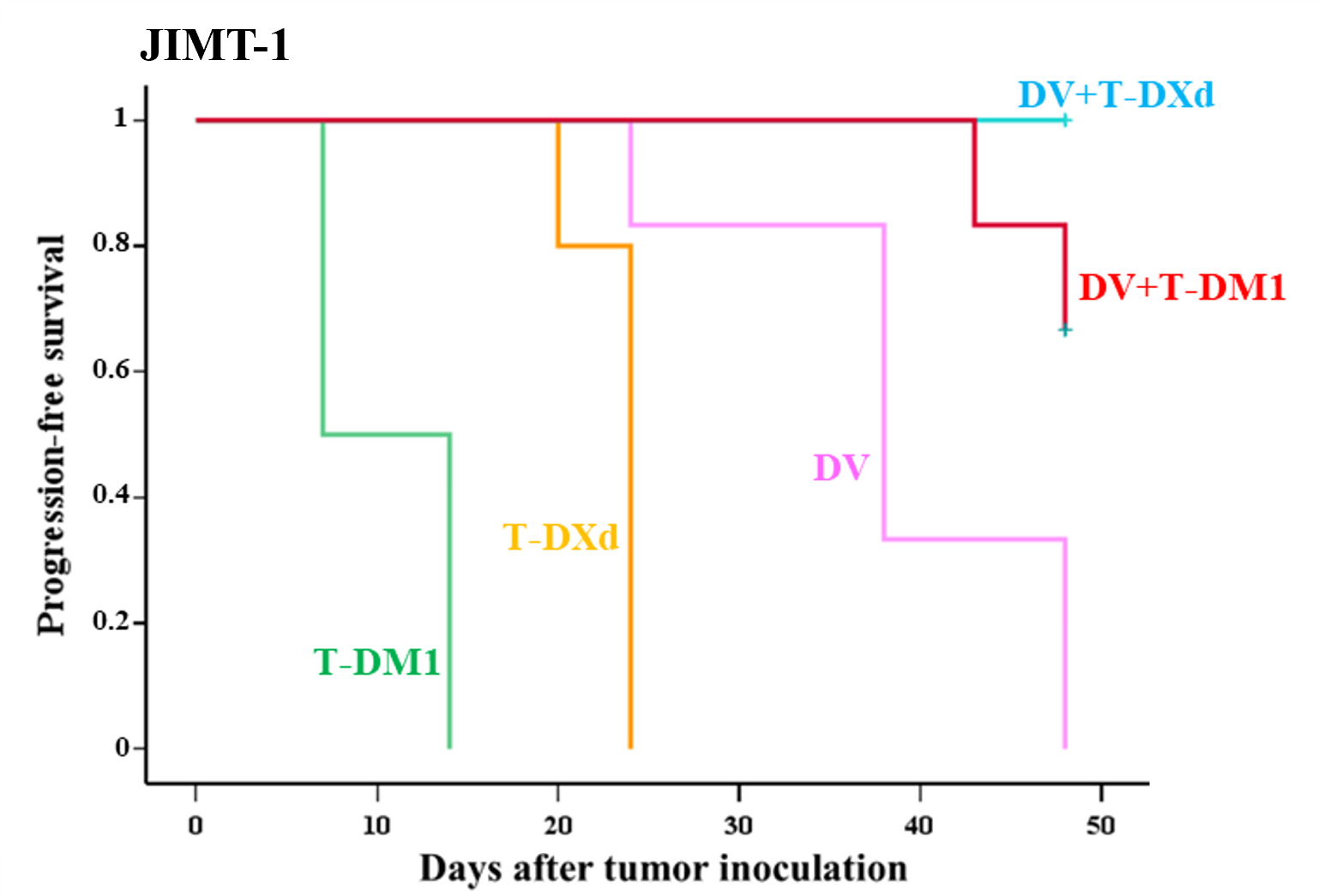

Supplement: Supplementary file 3 [file mmc3.jpg]

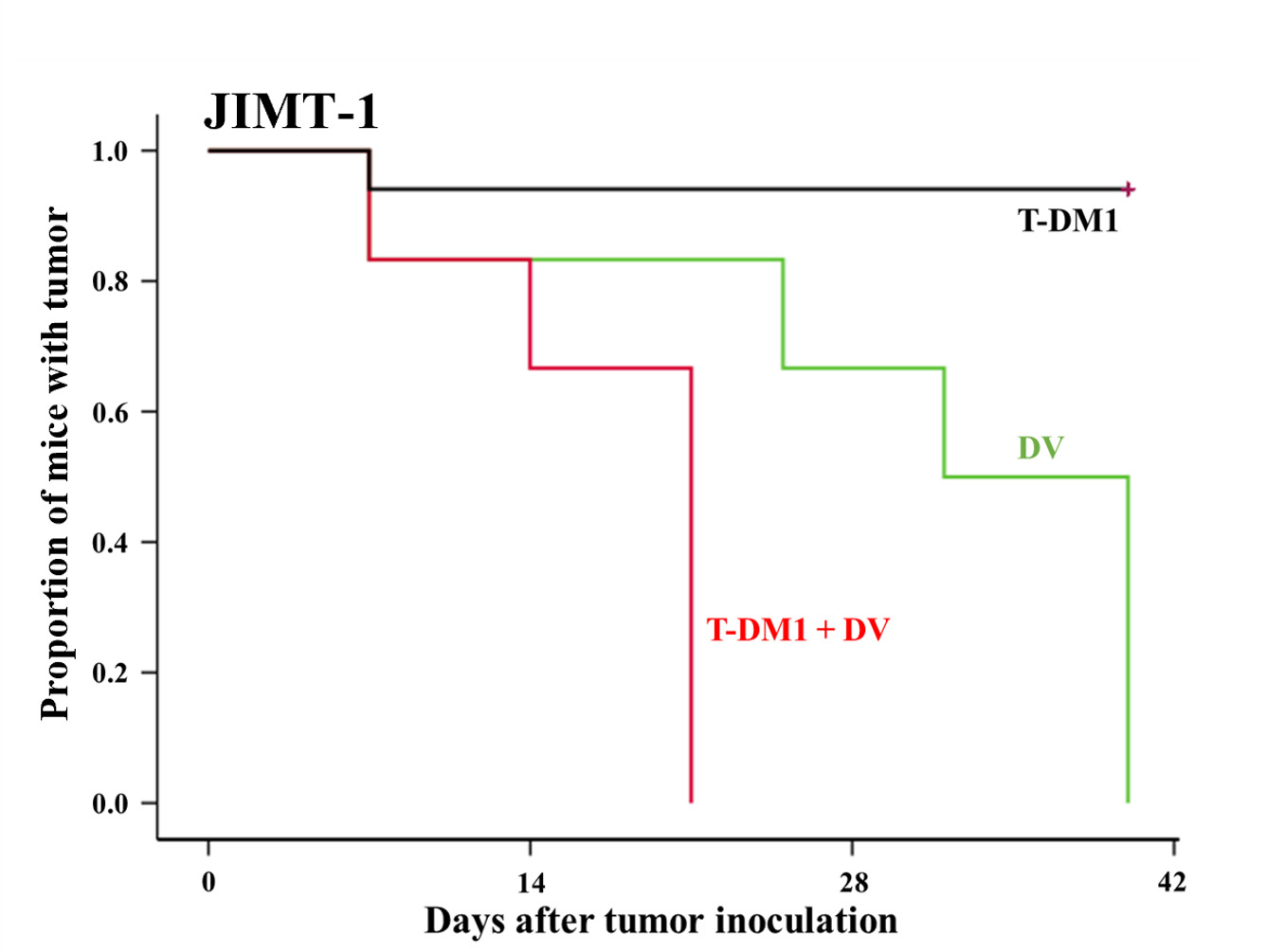

Supplement: Supplementary file 4 [file mmc4.jpg]

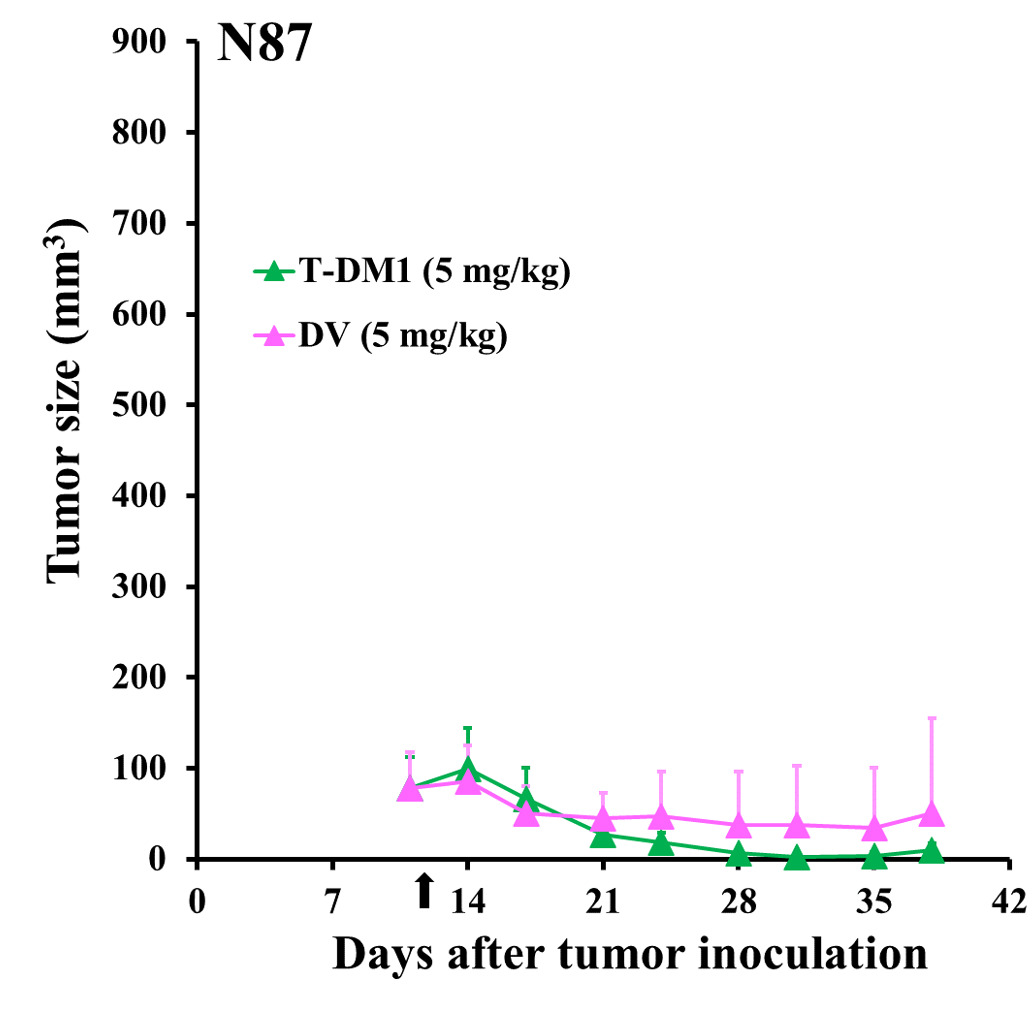

Supplement: Supplementary file 5 [file mmc5.jpg]

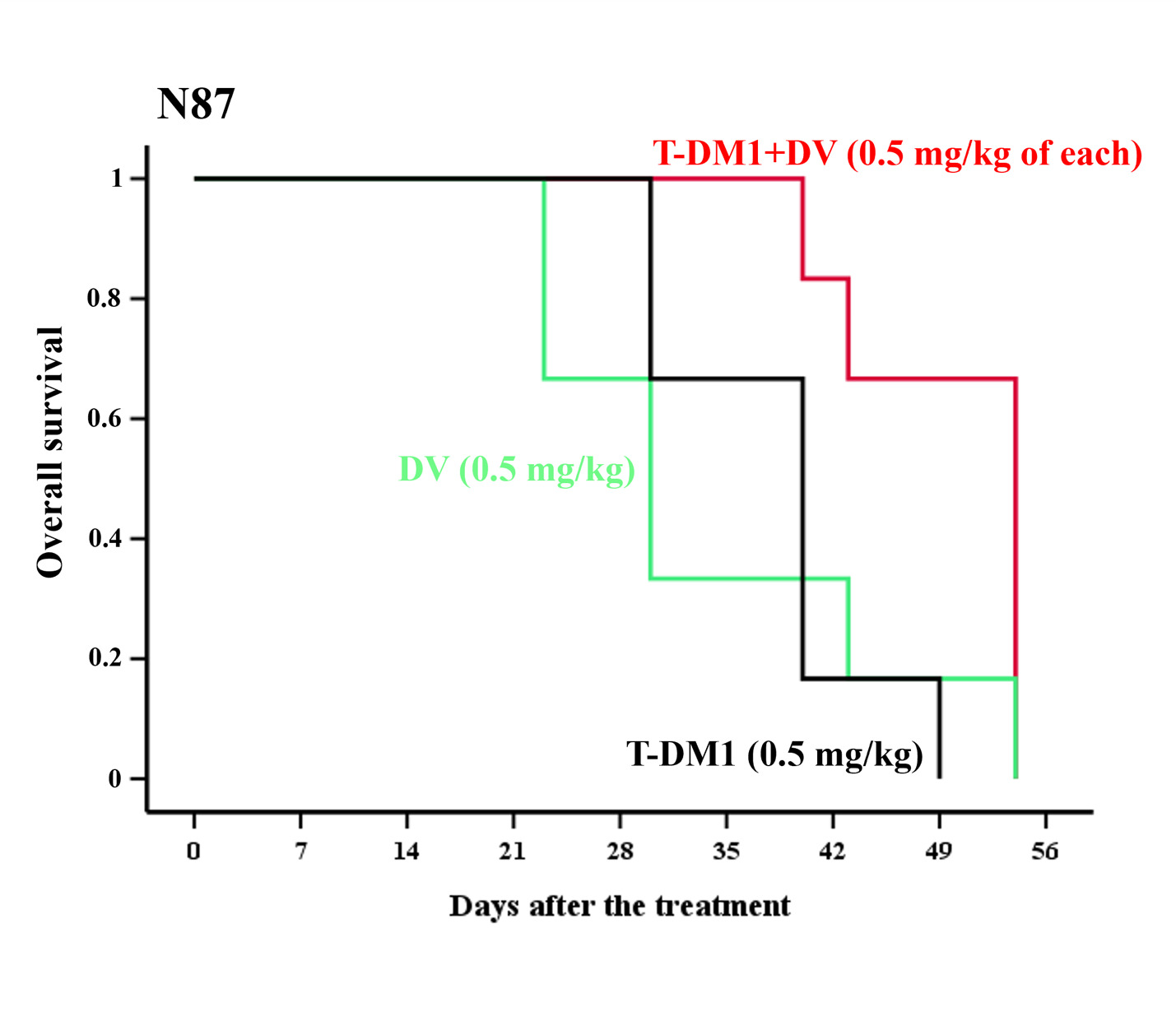

Supplement: Supplementary file 6 [file mmc6.jpg]

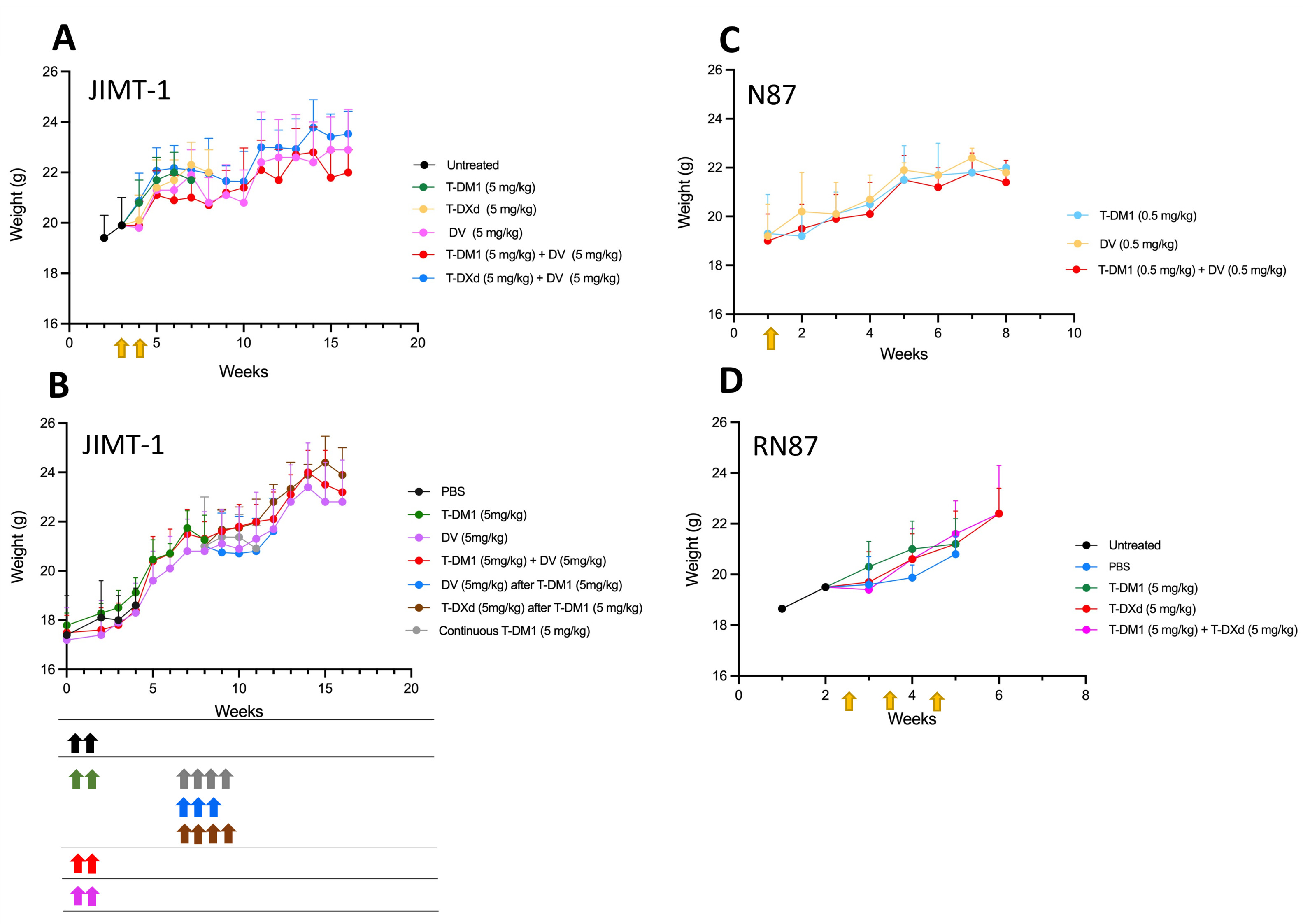

Supplement: Supplementary file 7 [file mmc7.jpg]

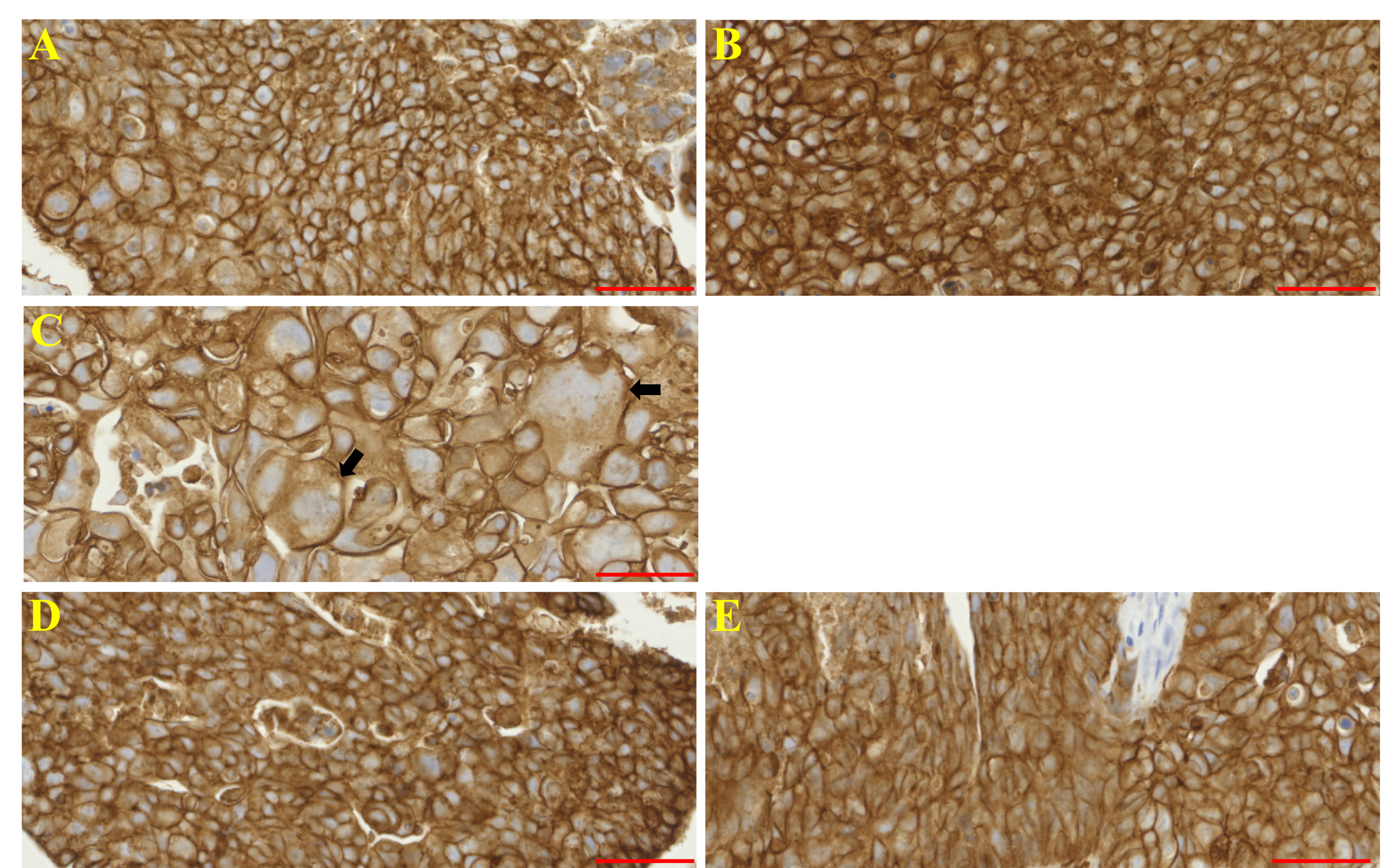

Supplement: Supplementary file 8 [file mmc8.jpg]

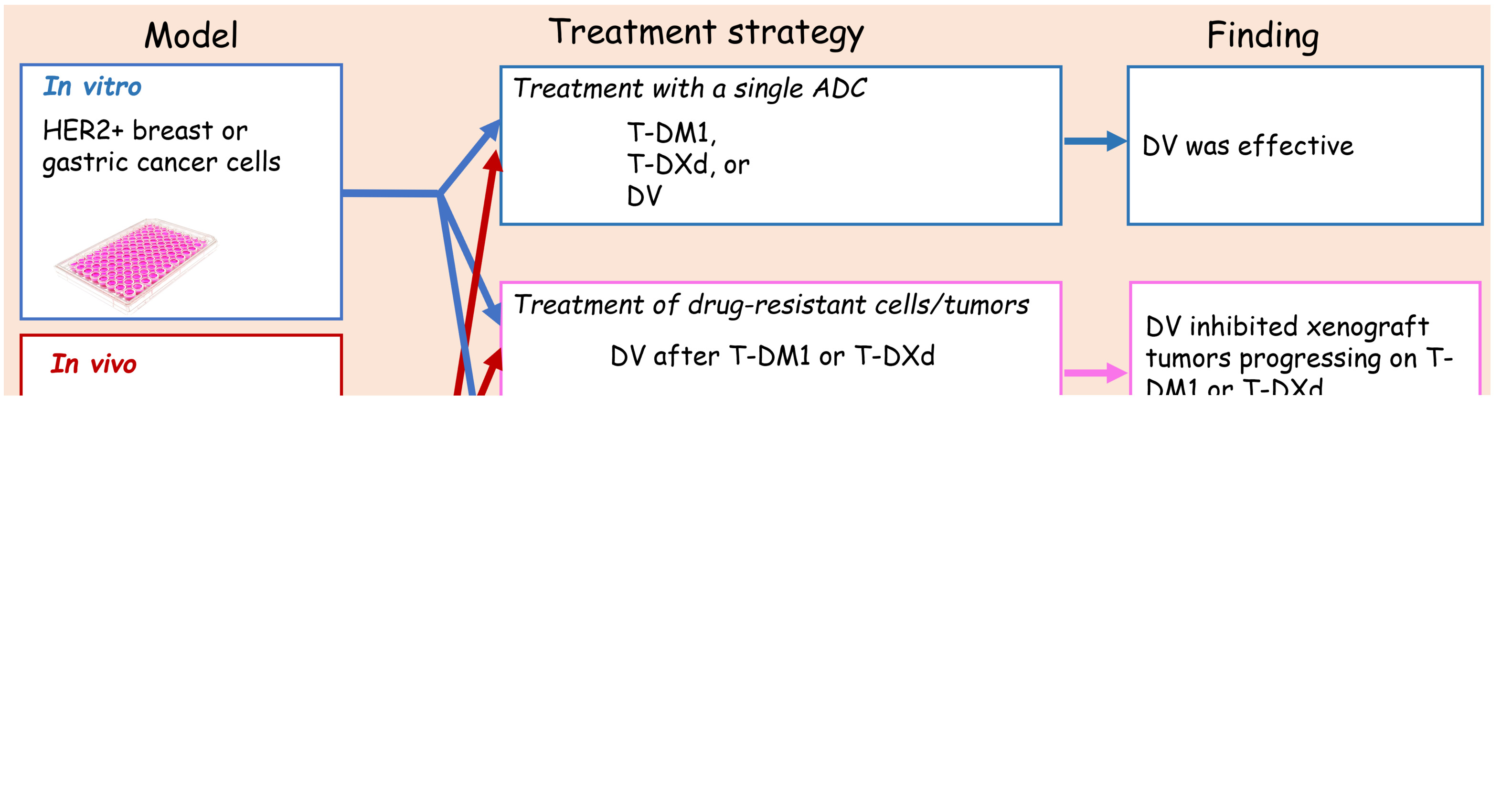

Supplement: Supplementary file 9 [file mmc9.jpg]
